# Matrix forming characteristics of inner and outer human meniscus cells on 3D collagen scaffolds under normal and low oxygen tensions

**DOI:** 10.1186/1471-2474-14-353

**Published:** 2013-12-13

**Authors:** Roger Croutze, Nadr Jomha, Hasan Uludag, Adetola Adesida

**Affiliations:** 1 Department of Surgery, Division of Orthopaedic Surgery, Laboratory of Stem Cell Biology and Orthopaedic Tissue Engineering, University of Alberta, Faculty of Medicine and Dentistry, 3.002E Li Ka Shing Centre for Health Research Innovation, Edmonton, AB T6G 2E1, Canada

**Keywords:** Meniscus, Meniscus fibrochondrocyte, Oxygen tension, Normoxia, Hypoxia, Collagen, Scaffold, Tissue engineering

## Abstract

**Background:**

Limited intrinsic healing potential of the meniscus and a strong correlation between meniscal injury and osteoarthritis have prompted investigation of surgical repair options, including the implantation of functional bioengineered constructs. Cell-based constructs appear promising, however the generation of meniscal constructs is complicated by the presence of diverse cell populations within this heterogeneous tissue and gaps in the information concerning their response to manipulation of oxygen tension during cell culture.

**Methods:**

Four human lateral menisci were harvested from patients undergoing total knee replacement. Inner and outer meniscal fibrochondrocytes (MFCs) were expanded to passage 3 in growth medium supplemented with basic fibroblast growth factor (FGF-2), then embedded in porous collagen type I scaffolds and chondrogenically stimulated with transforming growth factor β3 (TGF-β3) under 21% (normal or normoxic) or 3% (hypoxic) oxygen tension for 21 days. Following scaffold culture, constructs were analyzed biochemically for glycosaminoglycan production, histologically for deposition of extracellular matrix (ECM), as well as at the molecular level for expression of characteristic mRNA transcripts.

**Results:**

Constructs cultured under normal oxygen tension expressed higher levels of collagen type II (p = 0.05), aggrecan (p < 0.05) and cartilage oligomeric matrix protein, (COMP) (p < 0.05) compared to hypoxic expanded and cultured constructs. Accumulation of ECM rich in collagen type II and sulfated proteoglycan was evident in normoxic cultured scaffolds compared to those under low oxygen tension. There was no significant difference in expression of these genes between scaffolds seeded with MFCs isolated from inner or outer regions of the tissue following 21 days chondrogenic stimulation (p > 0.05).

**Conclusions:**

Cells isolated from inner and outer regions of the human meniscus demonstrated equivalent differentiation potential toward chondrogenic phenotype and ECM production. Oxygen tension played a key role in modulating the redifferentiation of meniscal fibrochondrocytes on a 3D collagen scaffold in vitro.

## Background

The medial and lateral menisci are C-shaped fibrocartilaginous wedges located between the femoral condyles and tibial plateau, which transmit 50-90% of load across the joint space [1]. These biomechanically crucial semilunar tissues play a role in joint stabilization, proprioception, lubrication and protection of the articular cartilage [2]. The ECM, responsible for maintaining structural and functional properties of the tissue, is synthesized entirely by cells within the tissue exhibiting both fibroblastic and chondrocytic characteristics, and referred to herein as meniscal fibrochondrocytes (MFCs). Fibroblast-like cells in the outer 1/3^rd^ of the tissue produce high amounts of collagen type I while chondrocyte-like cells, located in the inner 2/3^rd^, synthesize elevated levels of collagen type II and proteoglycan [3-5]. This geometrically and biochemically complex tissue is fully vascularized during fetal development though blood supply diminishes over time, receding to the peripheral 20-30% by 10 years of age [6,7].

Avascularity of the inner 2/3^rd^ of the meniscus is associated with limited healing potential of this zone [8,9], and presents a significant clinical problem considering the high incidence of meniscal injury [10,11]. Unfortunately, both traumatic injury and surgical resection of total or partial meniscal tissues correlate strongly with the progression of symptomatic osteoarthritis [12-15]. Recent understanding of this association promotes the sparing of meniscal tissue through arthroscopic partial meniscectomy alone, or in combination with treatment options including allograft transplantation [16], trephination [17], synovial debridement [18,19], and replacement of excised tissues with engineered constructs [20,21]. Meniscal allograft transplantation is limited by factors including graft rejection, donor shortage and risk of transmission of infectious disease [22]. Engineering of biologically-driven tissues provides a promising avenue for meniscal repair, though their implantation has only been compatible with partial meniscectomy to date [23]. Knowledge gaps concerning the cell physiology of MFCs and their response to culture microenvironments applied in tissue engineering remain to be investigated.

MFCs undergo morphologic and genotypic dedifferentiation in monolayer expansion similar to articular chondrocytes [24,25], which compounds the challenge of developing constructs mimicking the structural and biochemical properties of native meniscus. Scaffold culture has been investigated as a means to promote MFC redifferentiation following monolayer proliferation, based on the principle that native ECM is 3-dimensional and more closely approximates the *in vivo* environment of MFCs. These attempts to engineer musculoskeletal tissues involve the culture of select cell populations embedded in natural or polymer-based scaffolds, in the presence of a defined growth medium [26]. Early investigations regarding scaffold culture of MFCs documented that bovine MFCs successfully integrate and proliferate within a porous collagen matrix [27]. More recently, human MFCs have been expanded and cultured on 3D collagenous scaffolds [5,28,29] indicating the potential for cell-seeded tissue engineered constructs in allogeneic meniscal repair.

Previous work in the authors’ lab indicates that oxygen tension plays a significant role in modulating gene expression of MFCs cultured in a 3D environment and subsequently their capacity to synthesize abundant ECM rich in collagen type II and aggrecan [25,29]. In these studies, employing hypoxic (3%) oxygen tension during serial expansion and culture of MFC lead to increased mRNA transcript levels of matrix-associated proteoglycans fibromodulin and biglycan, as well as enhanced re-expression of collagen type II. These differences in gene expression and tissue production under varying parameters of oxygen tension can be attributed to the avascular nature of the inner meniscus and the hypoxic environment within the knee joint, where oxygen tension of the articular cartilage reaches 1-7% depending on tissue depth [30]. These studies were limited by the use of cells isolated from the meniscus as a whole, while a morphological and phenotypic distinction exists between fibroblast-like cells isolated from the outer vascular region of the tissue and chondrocyte-like cells isolated from the inner avascular zone [3-5]. Considering the dramatic effects of hypoxic culture on tissue production by MFCs, and in light of these limitations, further study concerning the response of unique MFC populations to differentiation under controlled 3D culture conditions of normal (21%) or low (3%) oxygen tension is merited.

In the present study, we examine the response of isolated inner and outer MFCs to growth factor supplemented culture on 3D porous collagen type I scaffolds. Our primary interest is in determining the differentiation potential of these cell populations under experimentally controlled parameters of oxygen tension. Given the inherent differences in vascularity [7], ECM composition [4] and distribution of cell population within native inner and outer meniscus tissue *in vivo*[3] detailed previously, we hypothesize that cells isolated from the outer meniscus will demonstrate a fibroblast-like genotype with elevated levels of collagen type I relative to collagen type II in response to scaffold culture, particularly under normoxic culture conditions. In contrast, we hypothesized that inner MFCs will express elevated levels of chondrocyte-like genetic markers, specifically high collagen type II, COMP and aggrecan along with low expression of collagen type I, with this effect being enhanced under low oxygen tension.

## Methods

### Ethics statement

Whole human menisci were harvested from patients undergoing total knee replacement surgery. Approval for this study was obtained from the ethics review board of the University of Alberta, Edmonton, Canada and safety guidelines were followed. Consideration was taken to preserve the privacy of specimen donors and the need for written informed consent was waived considering tissues were intended for discard in the normal course of surgical procedure.

### Isolation and monolayer expansion of MFCs

Human lateral menisci were obtained from four female patients undergoing total knee arthroplasty at the Royal Alexandra Hospital Orthopaedic Surgery Center in Edmonton, AB (N = 4, average age 66.8 years ± 4.4, range 54 – 79 years). Tissues appeared macroscopically normal, lacking the calcification and osteophyte formation characteristic of advanced osteoarthritic disease. Menisci were divided into separate outer 1/3^rd^ and inner 2/3^rd^ regions under aseptic conditions. MFCs were released from these sections by incubation for 1 hour at 37°C in trypsin-EDTA (0.05%; Mediatech Inc. VA, USA) followed by 22 hours at 37°C in type II collagenase (0.15% w/v; Worthington, Lakewood, NJ, USA) in an expansion medium composed of high glucose Dulbecco’s modified Eagle’s medium (DMEM; 4.5 mg/ml D-Glucose) supplemented with 5% fetal bovine serum, 2 mM L-glutamine, 100 units/ml penicillin and 100 units/ml streptomycin (Invitrogen) as previously detailed [25]. Isolated cells were plated at 10^4^ cells/cm^2^ and cultured in expansion medium previously defined, supplemented with FGF-2 (5 ng/ml; Humanzyme-Medicorp Inc., Montreal, Quebec, Canada), at 37°C under normoxia (21% O_2_) or hypoxia (3% O_2_) in a humidified incubator with 5% CO_2_. Media change was performed twice weekly in a normoxic environment, with media mixed and stored at 21% O_2_ prior to use, and taking care to limit the duration of exposure of hypoxic cultured MFCs to ambient oxygen. When cells reached sub-confluence, first passage (P1) cells were detached with trypsin-EDTA (0.05% w/v) and split 1:2 in order to produce passage 2 cells (P2) that were expanded for another doubling to produce a final monolayer culture of passage 3 (P3) cells. At P1 and P3, 2.5×10^5^ cells were transferred to 1 ml Trizol for gene expression analysis of tissue cultured MFCs through the course of monolayer proliferation.

### Scaffold seeding and construct culture

Porous collagen type I matrix derived from bovine Achilles tendon (10 cm × 12.5 cm; ~3.5 mm total thickness with pore size of 115 ± 20 μm; Integra Lifesciences, Plainsboro, NJ, USA) was biopsied into 6 mm diameter disks under sterile conditions. All disks were cut from the same batch of collagen scaffold in order to limit variability. Scaffolds were placed in a 24-well plate and seeded with 1×10^6^ MFCs per scaffold, suspended in 10 μl of a defined serum-free chondrogenic medium consisting of high glucose DMEM containing non-essential amino acids (0.1 mM), sodium pyruvate (1 mM), HEPES buffer (100 mM), L-glutamine (0.29 mg/ml), penicillin (100U/ml) and streptomycin (100U/ml) all from Invitrogen, supplemented with ascorbic acid 2-phosphate (0.1 mM), L-proline (40 ug/ml), dexamethasone (10^-5^ M), 1× ITS + 1 premix (Sigma-Aldrich, Oakville, Canada) and TGF-β3 (10 ng/ml; Humanzyme-Medicorp Inc.). Cell-seeded disks were incubated in a humidified incubator at 37°C, 21% O_2_ and 5% CO_2_ for 15 minutes to allow for initial cell attachment, then 100 μl of chondrogenic medium was carefully added to the base of each well prior to 30 minute incubation under the same conditions. Following the 30 minute incubation period, 1 ml of chondrogenic medium was gently added to cover each scaffold. Constructs loaded with MFCs expanded under normal oxygen tension (21% O_2_) were cultured for a total of 21 days at 37°C under 21% O_2_ and 5% CO_2_, while those seeded with MFCs expanded under low oxygen tension (3% O_2_) were cultured for 21 days at 37°C under 3% O_2_ and 5% CO_2_. Media change was performed twice per week, with complete replenishment of chondrogenic medium defined above. At the end of 3-day and 21-day scaffold culture periods, the engineered constructs were processed for gene expression analysis by real time quantitative reverse transcription polymerase chain reaction (qRT-PCR). Following 21-day scaffold culture, the remaining constructs were analyzed histologically and immunohistochemically for synthesis of ECM proteins and assayed biochemically for DNA and glycosaminoglycan (GAG) content.

### Histology

Day 21 (D21) engineered constructs were fixed overnight in 10% neutral buffered formalin at 4°C prior to histological processing and embedding in paraffin wax. 5 μm thickness sections were stained with 0.01% (w/v) Safranin-O and counterstained with 0.02% (w/v) Fast Green FCF in order to visualize accumulation of secreted sulfated proteoglycans. Other sections were probed for deposition of collagen type II by pretreatment with 0.1% (w/v) trypsin followed by incubation with monoclonal mouse antibodies against human collagen II at 1:50 dilution (II-II6B3; Developmental Studies Hybridoma Bank, University of Iowa, USA). Visualization of immunolocalized antigens was accomplished via incubation with goat anti-mouse IgG biotinylated secondary antibody in conjunction with a streptavidin-horseradish peroxidase labeling kit using 3,3′-diaminobenzidine as substrate (Dako Canada Inc., Mississauga, Ontario, Canada). Images were captured using an Optixcam Summit Series 5MP digital camera fitted to an Omano OM159T trinocular microscope (Microscope Store, Virginia, USA) using Optixcam software and assembled in Adobe Photoshop CS5 (Adobe Systems Inc., San Jose, USA).

### Biochemical analysis

Following 21-day culture in their respective conditions, scaffolds were rinsed in copious amounts of 1× phosphate buffered saline (Mediatech Inc.) in order to remove residual media then frozen at -80°C. Prior to biochemical assay, engineered constructs were digested in 500 μl Proteinase K (1 mg/ml in 50 mM Tris with 1 mM EDTA, 1 mM iodoacetamide and 10 mg/ml pepstatin A; all purchased from Sigma-Aldrich) overnight at 56°C. The content of sulfated GAG was quantified spectrophotometrically following addition of 1,9-dimethylmethylene blue using chondroitin sulfate as standard (Sigma-Aldrich) [31] with an MRX MicroPlate Reader (Dynatech Laboratories, Virginia, USA) measuring emissions at 520 nm. DNA quantitation was performed using CyQuant cell proliferation kit (Invitrogen) following manufacturer instructions, using supplied bacteriophage λ DNA as standard, and read at 530 nm on a CytoFluor II Fluorescence Multi-Well Plate Reader (PerSeptive Biosystems, Massachusetts, USA) following excitation at 450 nm.

### Isolation of genetic material

Total RNA was isolated from monolayer cultures at P1 and P3. 2.5×10^5^ cells were suspended in 1 ml Trizol (Invitrogen) and frozen at -80°C prior to RNA extraction using Aurum Total RNA Fatty and Fibrous Tissue Kit (BioRad, Mississauga, Ontario, Canada). Scaffold constructs were transferred to 1 ml Trizol following 3-day and 21-day culture periods and frozen at -80°C. RNA was extracted from scaffolds after mechanical disruption with grinding pestles, using a standard chloroform isopropanol extraction method following manufacturer’s instructions (Invitrogen). In order to mitigate changes in gene expression following removal from culture conditions, cells and scaffold constructs were transferred immediately to Trizol (less than 1 minute). The quantity and quality of isolated mRNA were assessed using a NanoDrop ND-1000 spectrophotometer (Thermo Scientific, Delaware, USA).

### Gene expression analysis by quantitative real-time Polymerase Chain Reaction (PCR)

Reverse transcription of total mRNA (100 ng) to cDNA was accomplished via GoScript reverse transcriptase using Promega cDNA synthesis kit (Fisher Scientific, Ontario, Canada) in a 40 μl reaction primed with random primers oligonucleotides. Real-time quantitative PCR was performed in a MJ Opticon I DNA Engine Continuous Fluorescence Detector (Bio-Rad, Ontario, Canada) using 1 μl cDNA in a 25 μl total reaction with hot start Taq polymerase and SYBR Green detection system (Eurogentec North America Inc., California, USA). Amplification occurred over a total of 40 cycles, ranging from 95°C to 60°C, with plate reading between denaturation and annealing steps. Primer sequences (Table 1) were based on previous work [25,32-34] and were purchased from Invitrogen (Mississauga, Ontario, Canada). mRNA expression levels for each gene were normalized to β-actin levels using the 2^-ΔCT^ method [35], where ΔCT = (threshold value of gene of interest) – (threshold value of β-actin).

**Table 1:** **Primer sequences used for real**-**time PCR analysis**

**Gene**	**Primer Sequence**	**Direction**	**Reference**
**Aggrecan**	**5**′**AGGGCGAGTGGAATGATGTT3**′	**Forward**	**32**
	**5**′**GGTGGCTGTGCCCTTTTTAC3**′	**Reverse**	
**COL1A2**	**5**′**TTGCCCAAAGTTGTCCTCTTCT3**′	**Forward**	**32**
	**5**′**AGCTTCTGTGGAACCATGGAA3**′	**Reverse**	
**COL2A1**	**5**′**CTGCAAAATAAAATCTCGGTGTTCT3**′	**Forward**	**32**
	**5**′**GGGCATTTGACTCACACCAGT3**′	**Reverse**	
**COMP**	**5**′**GCGAAACGTGGGTTGGAA3**′	**Forward**	**36**
	**5**′**GCCGGTGCTGCAGGAA3**′	**Reverse**	
**HIF**-**1α**	**5**′**GTAGTTGTGGAAGTTTATGCTAATATTGTGT3**′	**Forward**	**34**
	**5**′**CTTGTTTACAGTCTGCTCAAAATATCTT3**′	**Reverse**	
**HIF**-**2α**	**5**′**GGTGGCAGAACTTGAAGGGTTA3**′	**Forward**	**35**
	**5**′**GGGCAACACACACAGGAAATC3**′	**Reverse**	
**Sox9**	**5**′**CTTTGGTTTGTGTTCGTGTTTTG3**′	**Forward**	**32**
	**5**′**AGAGAAAGAAAAAGGGAAAGGTAAGTTT3**′	**Reverse**	
**β**-**actin**	**5**′**AAGCCACCCCACTTCTCTCTAA3**′	**Forward**	**32**
	**5**′**AATGCTATCACCTCCCCTGTGT3**′	**Reverse**	

All primers were purchased from Invitrogen (Mississauga, Ontario, Canada). COL1A2, collagen type I α2 chain; COL2A1, collagen type II α1 chain; COMP, cartilage oligomeric matrix protein; HIF-1α, hypoxia-inducible factor 1α; HIF-2α, hypoxia-inducible factor 2α; Sox9, Sry-related HMG box 9.

### Statistical analysis

Data for monolayer P1 and P3 cells represent the mean ± standard error of the mean of a single sampling of cell cultures collected from four independent donors (N = 4, n = 4). Data for GAG/DNA assay represent mean ± standard error of the mean of a total of four independent experiments conducted in duplicate (N = 4, n = 8). Data for qRT-PCR are presented as mean ± standard error of the mean of a minimum of 2 and maximum of 3 replicates of measurements acquired from 4 donor specimens (N = 4, n = 10). Statistically significant differences between groups were determined via Student’s t-test using Microsoft Excel. Statistical differences between multiple groups were assessed by one-way ANOVA and adjusted by Games-Howell multiple comparisons post-tests using SPSS (version 20). All differences were considered statistically significant at p < 0.05.

## Results

### A. Gene expression

#### MFCs undergo dedifferentiation and loss of phenotypic markers over the course of monolayer expansion

**Figure 1:**
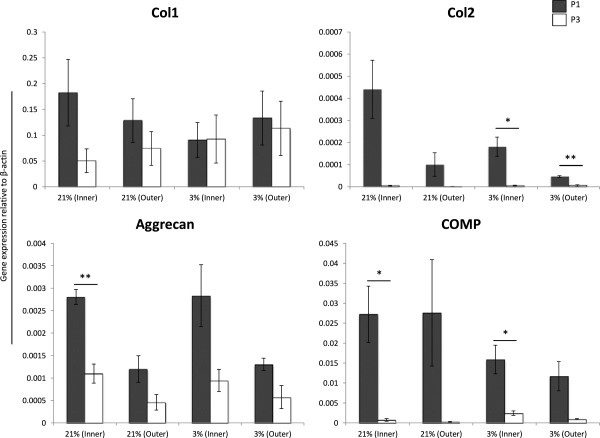
**Phenotypic loss of fibrocartilaginous markers during monolayer expansion.** Inner and outer MFCs were expanded from Passage 1 (P1, grey bars) to Passage 3 (P3, white bars) under normal (21%) or low (3%) oxygen tension. mRNA transcript levels of collagen type I (Col1), collagen type II (Col 2), aggrecan and COMP were compared within groups at P1 and P3. Marked dedifferentiation is seen between P1 and P3 for all genes of interest except collagen type I. Expression was normalized to levels of the housekeeping gene β-actin using 2^-ΔCT^ method. * = p < 0.05 ** = p < 0.01 represents significant difference between P1 and P3 values within a single group using Student’s two-tailed t-test.

**Figure 2:**
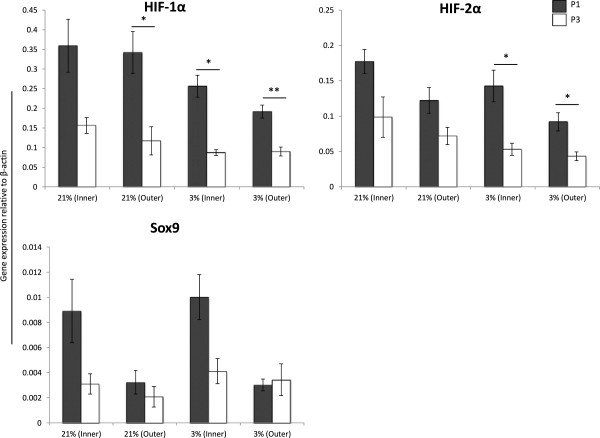
**Phenotypic loss of key transcription factors during monolayer expansion.** Inner and outer MFCs were expanded from Passage 1 (P1, grey bars) to Passage 3 (P3, white bars) under normal (21%) or low (3%) oxygen tension. mRNA transcript levels of hypoxia-inducible factor 1α (HIF-1α), hypoxia-inducible factor 2α (HIF-2α) and Sry-related HMG box-9 (Sox9) were compared within groups at P1 and P3. Marked dedifferentiation is seen between P1 and P3 for all genes of interest except Sox9. Expression was normalized to levels of the housekeeping gene β-actin using 2^-ΔCT^ method. * = p < 0.05 ** = p < 0.01 represents significant difference between P1 and P3 values within a single group using Student’s two-tailed t-test.

mRNA transcript levels of collagen type II, aggrecan, COMP, hypoxia inducible factor 1α and 2α show marked decline between Passage 1 (P1) and Passage 3(P3) of serial monolayer expansion (Figures 1 and 2). Collagen type II expression, a hallmark of chondrocytic lineage, is significantly depressed following monolayer expansion under hypoxic conditions, and approaches significance in the group of inner MFC cultured under normal oxygen tension (p = 0.06). Genetic expression levels of collagen type I (Figure 1) and Sox9 (Figure 2) remain low, with insignificant difference between P1 and P3 values.

### MFCs express elevated levels of fibrochondrogenic markers following chondrogenic stimulation in scaffold culture

The chondrogenic differentiation potential of inner 2/3^rd^ and outer 1/3^rd^ human MFCs cultured in collagen type I scaffolds for 21 days under normal (21%) or low (3%) oxygen tension was determined at the molecular level by comparing gene expression of monolayer cells immediately prior to cell seeding (P3) with scaffold constructs at the end of chondrogenic culture (D21). In most instances, expression of collagen types I and II, aggrecan, COMP and Sox9 were significantly higher in D21 scaffold constructs compared to P3.

**Figure 3:**
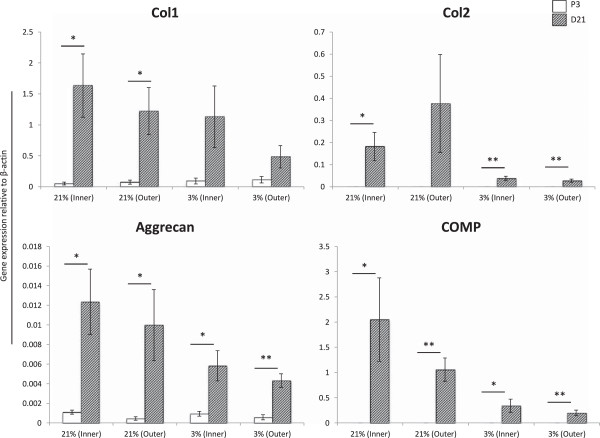
**Expression of fibrocartilaginous markers at P3 versus D21.** Inner and outer MFCs were expanded to Passage 3 (P3, white bars) then chondrogenically stimulated in scaffold culture under 21% or 3% O_2_ in vitro for 21 days (D21, hatched bars). mRNA transcript expression levels were analyzed for collagen type I (Col1), collagen type II (Col2), aggrecan and cartilage oligomeric matrix protein (COMP). Expression was normalized to levels of the housekeeping gene β-actin using 2^-ΔCT^ method. * = p < 0.05 ** = p < 0.01 represents significant difference between P3 and D21 values within groups using Student’s two-tailed t-test. There was no significant difference in expression levels of these mRNA transcripts between D21 scaffold constructs from inner or outer MFCs cultured under 21% or 3% oxygen tension. # = p <0.05 by one-way ANOVA with Games-Howell multiple comparisons post-test.

Following 21 days scaffold culture period (D21), expression of the collagen type I (*col1a2*) mRNA transcript was significantly elevated in both inner and outer normoxic cultured constructs compared to P3 values. This difference between P3 and D21 cultures was not significant for hypoxic cultured constructs (Figure 3).

Expression levels of the early developmental form of collagen type II (*Col2a1*) were also higher after 3D culture (D21) compared to monolayer expansion (P3). These differences were significant in three of the four culture conditions (Figure 3).

Aggrecan expression was significantly higher in D21 scaffolds relative to P3 monolayer cells in all four conditions by 11.2-, 21.7-, 6.2- and 7.5-fold for inner normoxic, outer normoxic, inner hypoxic and outer hypoxic constructs, respectively (Figure 3).

Cartilage oligomeric matrix protein (*COMP*) expression levels were also significantly elevated at the end of 21 days scaffold culture in all four conditions. The differences were 2700-, 3900-, 138- and 209-fold higher at D21 compared to P3 for inner normoxic, outer normoxic, inner hypoxic and outer hypoxic constructs, respectively (Figure 3).

**Figure 4:**
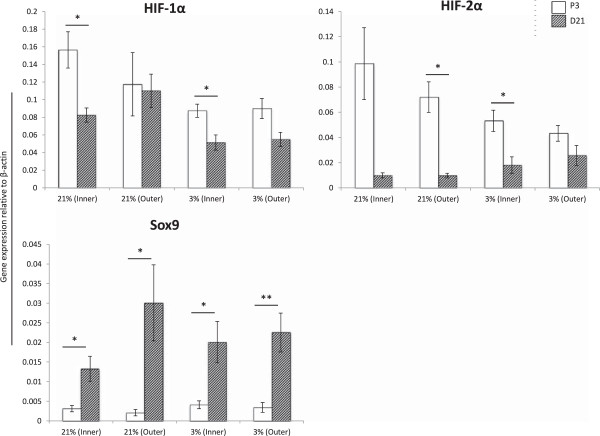
**mRNA transcript levels of key transcription factors at P3 versus D21.** Expression levels of mRNA transcript were analyzed for transcription factors hypoxia-inducible factor 1α (HIF-1α), hypoxia-inducible factor 2α (HIF-2α) and Sry-related HMG box-9 (Sox9) at the end of P3 monolayer expansion (P3, white bars) compared to 21 days scaffold culture (D21, hatched bars). Scaffold culture increased Sox9 expression in all culture conditions. Gene expression was normalized to levels of the housekeeping gene β-actin using 2^-ΔCT^ method. * = p < 0.05 represents significant difference between P3 and D21 values within groups using Student’s two-tailed t-test. There was no significant difference in expression levels of these mRNA transcripts between D21 scaffold constructs from inner or outer MFCs cultured under 21% or 3% oxygen tension. # = p <0.05 by one-way ANOVA with Games-Howell multiple comparisons post-test.

Surprisingly, in contrast to the other transcripts probed, there was decreased expression of both HIF-1α and HIF-2α in D21 scaffolds constructs compared to monolayer cells prior to chondrogenic stimulation (Figure 4).

Like aggrecan and COMP, expression of *Sox9* was elevated in D21 scaffold constructs by 4.3-, 14.4-, 4.9-, and 6.6-fold compared to P3 monolayer cells in 21% inner, 21% outer, 3% inner and 3% outer culture conditions, respectively. These differences were statistically significant (Figure 4).

### Inner and outer MFCs demonstrate equivalent redifferentiation potential under chondrogenic stimulation

The molecular expression of characteristic fibrochondrogenic markers was evaluated in D21 scaffold constructs generated under four culture conditions: inner MFCs cultured under normal (21% O_2_) and low oxygen tension (3% O_2_) as well as outer MFCs cultured under the same two oxygen tensions. Interestingly, there was no significant difference (p > 0.05) in expression of collagen types I or II, aggrecan, or COMP between these four groups by one-way ANOVA (Figure 3).

Differences in the expression of transcription factors Sox9, HIF-1α and HIF-2α in D21 scaffold constructs assessed by one-way ANOVA were also not significant between culture conditions (p > 0.05) (Figure 4).

### In vitro MFC differentiation is enhanced by culture under normal oxygen tension

**Figure 5:**
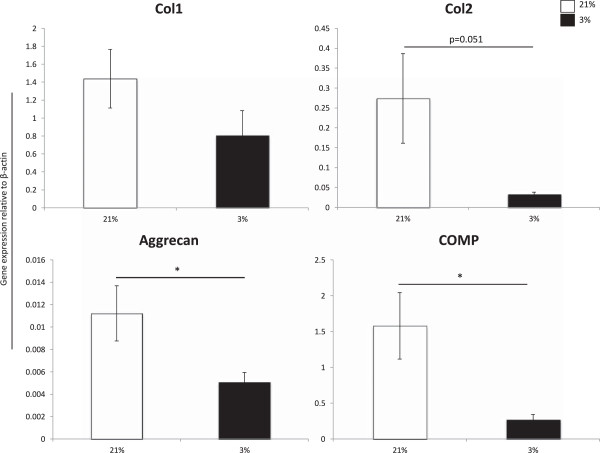
**Gene expression levels of D21 scaffolds cultured under normal versus low oxygen tension.** Inner and outer MFCs were expanded to P3 then chondrogenically stimulated in scaffold culture under normal (21%, white bars) or low (3%, black bars) O_2_ in vitro for 21 days (D21). Data represents the mean ± SEM of pooled values for inner and outer MFC constructs cultured under 21% compared to those cultured under 3% oxygen tension. Expression levels were normalized to β-actin using 2^-ΔCT^ method. * = p < 0.05 using Student’s two-tailed t-test.

In order to assess the influence of oxygen tension on differentiation of MFCs in scaffold culture models *in vitro*, mRNA transcript levels of D21 scaffold constructs seeded with inner and outer MFCs cultured under normoxia were pooled and compared to levels observed in all constructs cultured under hypoxia. There was no significant difference in expression of collagen type I (*col1a2*) between oxygen tensions. In contrast, normoxic cultured constructs expressed the collagen type II transcript (*col2a1*) at an evidently elevated level approaching statistical significance (p = 0.05) compared to hypoxic cultured constructs (Figure 5). Interestingly, expressions of aggrecan and COMP were also significantly higher in normoxic cultured scaffolds compared to those generated under hypoxic culture (Figure 5). Normoxic cultured constructs expressed 8.5-, 2.2-, and 5.8-fold higher transcript levels of collagen type II, aggrecan and COMP, respectively.

**Figure 6:**
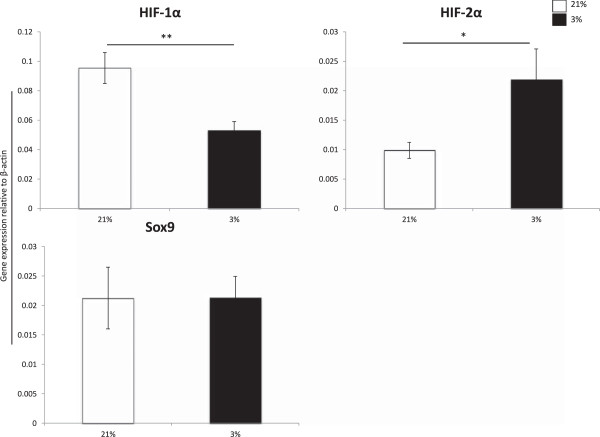
**Gene expression levels of D21 scaffolds cultured under normal versus low oxygen tension.** Inner and outer MFCs were expanded to P3 then chondrogenically stimulated in scaffold culture under normal (21%, white bars) or low (3%, black bars) O_2_ in vitro for 21 days (D21). Data represents the mean ± SEM of pooled values for inner and outer MFCs cultured under 21% compared to those cultured under 3% oxygen tension. Expression levels were normalized to β-actin using 2^-ΔCT^ method. * = p < 0.05 using Student’s two-tailed t-test.

Surprisingly, hypoxia inducible factor-1α (HIF-1α) expression was significantly higher in cells cultured under normoxia. In contrast, hypoxia inducible factor-2α (HIF-2α) was statistically elevated in hypoxic cultured scaffolds compared to those stimulated under normoxia (Figure 6).

Oxygen tension did not appear to modulate expression of Sox9 in scaffold culture, as there was no significant difference in transcript expression levels between scaffolds cultured at 21% oxygen versus 3% oxygen tension (Figure 6).

### B. Histology

#### Collagen II immunohistochemistry

**Figure 7:**
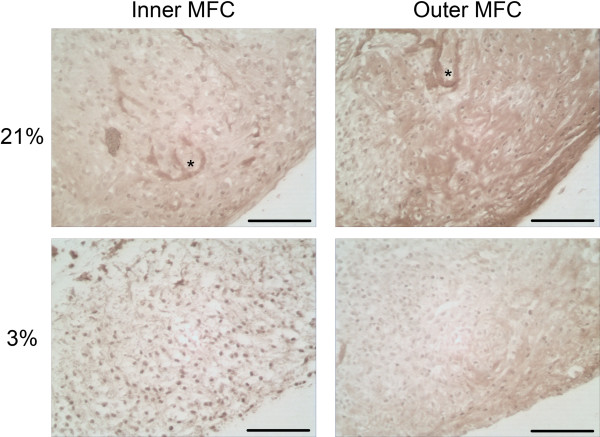
**Immunohistochemical staining for collagen II at D21.** Paraffin embedded sections of day 21 (D21) scaffold culture constructs were sectioned at 5 μm and the deposition of collagen type II was visualized by indirect immunohistochemistry in conjunction with streptavidin-horseradish peroxidase linked secondary antibody. Representative images of collagen type II distribution were captured from inner and outer MFCs cultured in 21% O_2_ and 3% O_2_ (scale bar 100 μm). Asterisk represents scaffold collagen fiber and surrounding area where fibers have been replaced by ECM synthesized de novo.

**Figure 8:**
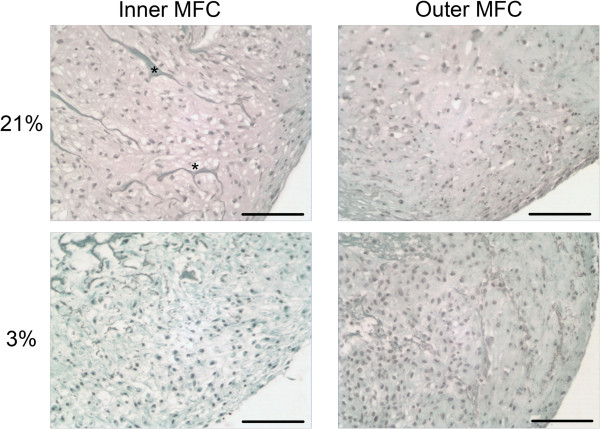
**Deposition of sulfated proteoglycan in D21 constructs.** Inner and outer MFC were cultured on collagen scaffolds under normal (21%) or low (3%) oxygen tension for 21 days. 5 μm paraffin-embedded sections were stained with Safranin-O and counterstained with fast green. Images captured from constructs seeded with inner and outer MFC cultured under normal (21% O_2_, top row) or low oxygen tension (3% O_2_, bottom row). Scale bar 100 μm. Asterisk represents scaffold collagen fiber and surrounding area where fibers have been replaced by ECM synthesized de novo.

Collagen scaffolds were seeded with MFCs isolated from inner and outer regions of the meniscus and cultured in chondrogenic medium for a total of 21 days under normal or low oxygen tensions. Scaffold constructs generated from MFCs expanded under normal oxygen tension were subsequently cultured under normoxia, while those generated from hypoxia expanded cells were then cultured under the same low oxygen tension. At the end of 21 days scaffold culture, deposition of collagen type II was assessed by indirect immunohistochemistry. MFCs appeared integrated with the porous collagen type I scaffold, remodeling the scaffold fibers while synthesizing tissue *de novo* (asterisk, Figures 7 and 8). The porous, peripheral surface of the cell-laden scaffolds where cells were loaded was populated with cells, though they did not permeate the full thickness of the scaffold and large acellular regions with little or no tissue production are present near the base. Synthesis of an ECM rich in collagen type II was demonstrated in constructs seeded with inner MFCs cultured under normal oxygen tension, as well as in tissues generated from outer MFCs cultured under the same normal (21%) oxygen tension. In contrast, scaffolds containing either cell type cultured under low (3%) oxygen tension demonstrated lower density and limited accumulation of collagen type II compared to normoxic cultured constructs (Figure 7). MFCs near the periphery of the scaffold constructs appeared sparse, had rounded chondrocyte-like morphology and were surrounded by defined lacunae with abundant interterritorial staining for collagen type II. Within the body of the construct cells were also rounded, though cell density was significantly higher and staining for collagen type II less abundant (Figure 7).

#### Safranin-O

The deposition and distribution of sulfated proteoglycan in the ECM of D21 scaffold constructs was visualized by Safranin-O staining of 5 μm thickness paraffin embedded sections. Strong positive staining was observed in scaffolds embedded with inner meniscus cells chondrogenically stimulated under normal oxygen tension as indicated by pink staining. In contrast, constructs generated from inner meniscus cells under low oxygen tension did not demonstrate any positive staining for sulfated proteoglycan. Scaffolds seeded with outer MFCs cultured under 21% oxygen tension demonstrated moderate Safranin-O staining while limited deposition of sulfated proteoglycan was noted in outer MFCs cultured under 3% oxygen tension. Meniscal fibrochondrocytes within the tissue sections had a rounded, chondrocyte-like morphology and appeared integrated with the scaffold (Figure 8).

### C. Biochemical analysis

**Figure 9:**
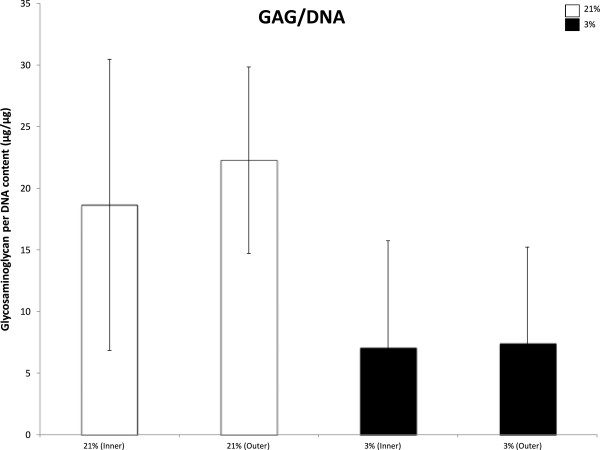
**Biochemical analysis of glycosaminoglycans in D21 scaffold constructs.** Mean GAG/DNA (μg/μg) levels from a total of four independent scaffold culture experiments performed in duplicate. MFCs isolated from inner and outer human meniscus were seeded on porous collagen scaffolds and chondrogenically stimulated under normal (21% O_2_, NRX) or low (3% O_2_, HYP) oxygen tension. Data represents mean ± standard error of the mean. * = p <0.05 using one-way ANOVA with Tukey’s multiple comparisons post-test.

To assess the differentiation potential of chondrogenically stimulated MFCs isolated from the inner and outer regions under normal or low oxygen tension, biochemical assay measuring the quantity of glycosaminoglycan produced per equal weight of genomic DNA (GAG/DNA) were conducted on D21 scaffold constructs. When considering pooled mean values from four independent experiments, there was no significant difference in GAG/DNA levels between cells isolated from inner and outer meniscus under either oxygen tension (Figure 9).

## Discussion

Over the course of monolayer expansion, the ability of cells to proliferate and maintain their characteristic differentiated phenotype is diminished [24,25]. MFCs undergo a documented change in morphology towards a predominantly elongated, fibroblast-like appearance along with a simultaneous down-regulation in expression levels of collagen type II and up-regulation of collagen type I mRNA transcripts [5,24]. Additionally, the genotypic and phenotypic expression patterns of MFCs may be altered or compromised by the disease process of osteoarthritis, which is a factor with the tissue used in this study. Considering that expansion is necessary in order to secure adequate cell numbers for the generation of engineered constructs, and that cells may be derived from osteoarthritic autogenous tissues, determining the appropriate culture conditions required to stimulate redifferentiation toward ECM production and phenotype characteristic of fibrocartilage is of paramount importance.

Our results disproved the original hypothesis. Rather than observing unique responses to culture under normal or low oxygen tensions by distinct inner and outer MFC populations, we report equivalent redifferentiation potential of inner and outer MFCs. Normoxic culture favored the genetic expression and protein synthesis of fibrochondrogenic markers including collagen type II, aggrecan and COMP in cells isolated from both inner and outer regions compared to those scaffolds incubated under hypoxic conditions.

Our observation that inner and outer MFCs are equally capable of redifferentiation toward a pattern of gene expression and ECM production recapitulating fibrocartilaginous tissue is consistent with previous work conducted by Mauck et al. [26]. Their investigations highlighted the plasticity of serially expanded MFCs, demonstrating that dedifferentiated cells from both inner and outer regions of the bovine meniscus could be stimulated toward a diversity of mesenchymal lineages including fibrocartilage, adipose tissue and bone [36]. We build from their work and show that given appropriate culture conditions, serially expanded human MFCs from both regions also possess equivalent redifferentiation capacity toward a fibrocartilaginous lineage.

Expansion media exerts a significant influence on the processes of cellular dedifferentiation and redifferentiation [25,37]. Accordingly, formulations of expansion medium and chondrogenic medium used in this study were carefully chosen. In a previous study, FGF-2 mediated monolayer expansion of meniscal cells promoted a 200-fold increase in collagen type II production and enhanced proteoglycan deposition in pellet culture, compared to controls [25]. This FGF-2 mediated potentiation of chondrogenic capacity, along with enhancement of proliferation rates and expedition of phenotypic loss, has also been demonstrated with similar findings in articular chondrocytes [37,38]. Chondrogenic culture in the presence of dexamethasone is associated with increased expression of Sox9 [39], an important transcriptional regulator at collagen type II and aggrecan promoter/enhancer regions [40]. Transforming growth factor beta (TGF-β) is known to up-regulate expression and deposition of collagen type II and proteoglycan in monolayer and scaffold cultures [5,28]. The effects of these growth supplements must be considered in interpreting the results of this study.

In the present study, we noted decreased mRNA transcript levels of collagen type II, aggrecan, COMP, Sox9, HIF-1α and HIF-2α over the course of monolayer expansion from P1 to P3. The loss of fibrocartilaginous phenotype is consistent with reports of genetic and morphologic changes in serially sub-cultivated MFCs [5,24] as well as chondrocytes [38]. However, up-regulation of collagen type I was not evident in the present study, where we instead report maintenance of low levels of collagen type I expression.

When embedded on a collagen scaffold and chondrogenically stimulated, MFCs expressed significantly increased mRNA transcript levels of fibrochondrogenic markers compared with monolayer (P3) cells immediately prior to scaffolding. Scaffolds incubated under normal oxygen tension expressed higher levels of collagen type II, aggrecan and COMP than scaffolds cultured under low oxygen tension. In addition to these findings at the molecular level, histological findings demonstrated that scaffolds cultured under normal oxygen tension deposited ECM most abundant in collagen type II and proteoglycan. These findings are consistent with genetic and histological findings in scaffolding experiments previously conducted in our lab demonstrating that human MFCs isolated from the entire meniscal tissue produce higher amounts of collagen type II under normoxic compared to hypoxic culture [29]. However, the previous study did not examine the differentiation potential of cells isolated from distinct inner and outer meniscal regions. The mechanisms by which normoxic culture of MFCs enhances transcript expression of chondrogenic markers collagen type II, aggrecan and COMP in scaffold culture *in vitro* remain to be elucidated. While interpreting these results, it is important to consider the role of the collagen scaffold, which is not inert, and may play a role in chemical and mechanical modulation of gene expression for example through integrin binding and mechanotransduction.

While previous work provides evidence for the involvement of Sox9 in transcriptional regulation of collagen type II and aggrecan in chondrocytes [40,41], in the present study we found no correlation between mRNA expression levels of Sox9 and collagen type II or aggrecan in re-differentiated MFCs. *In vivo* analysis of the relationship between Sox9 and collagen type II in articular chondrocytes [42], as well as gene expression analysis of scaffold constructs generated from MFCs [29] are consistent with our observation of a lack of correlation between transcript levels of Sox9 and collagen type II. In light of these findings, we suspect that while the presence of Sox9 is necessary at the enhancer/promoter region for transcription of collagen type II and aggrecan, concentration of the regulatory molecule above a threshold level has no influence in modulating expression of either transcript. Additionally, there are likely alternate molecular pathways implicated in the expression and synthesis of these important ECM components. Nonetheless, our data suggests that Sox9 is involved in chondrogenic redifferentiation of MFCs, considering expression of this gene increased significantly in chondrogenically stimulated D21 scaffolds compared to P3 monolayer cells in all four culture conditions.

An interesting finding in this study was the decreased expression of HIF-1α under hypoxic (3%) compared to normoxic (21%) oxygen tension, considering previous work in our lab demonstrated increased expression of HIF-1α and Sox9 in meniscus cells cultured under hypoxia [32]. As expected, HIF-2α transcript expression was significantly higher in constructs cultured under hypoxic culture conditions. Hypoxia inducible factors HIF-1α and HIF-2α are known to promote synthesis of cartilaginous ECM in chondrocytes through induction of the Sox9 pathway [43,44].

Considering the non-significant difference in genetic expression of aggrecan mRNA transcript between inner and outer MFC cultured under 21% or 3% O_2_, the results of our GAG/DNA assay demonstrating equivalent production of matrix-associated proteoglycan between groups of oxygen tension are not surprising. Overall production of proteoglycans was low compared to expected values, which could be explained by the fact that the MFC used in this study were taken from osteoarthritic joints. While no significant difference was noted in biochemical assay, histological evaluation of the deposition of sulfated proteoglycan in the ECM correlates with trends observed in biochemical analysis, with normoxic cultured scaffolds staining slightly more intensely than hypoxic cultured scaffolds.

The results of this study are limited by the use of tissue from patients with arthritic symptoms undergoing total knee replacement rather than normal menisci unaffected by disease, however precautions were taken only to harvest menisci with limited osteoarthritic changes appearing macroscopically normal. Additionally, the use of cells isolated from four independent donors presented significant inter-donor variability. With a larger sample size and reduced error margins, differences which were not statistically significant may have approached or reached statistical significance. Finally, isolating distinct cell populations is technically unfeasible and we instead relied upon anatomical proportions in order to separate inner and outer meniscal fibrochondrocyte populations.

This is the first study investigating the response of human inner and outer MFCs to normal and low oxygen tension within a collagenous 3D microenvironment in vitro. Taken together, our findings indicate that cells isolated from both the inner avascular and outer vascular regions of the meniscus demonstrate comparable levels of plasticity toward a fibrochondrogenic lineage following monolayer expansion and accompanying dedifferentiation. Meniscus cells embedded in 3D collagen scaffolds are most effectively stimulated to express and produce chondrocyte-like tissues, abundant in collagen type II and proteoglycan, when cultured under normal oxygen tension. The results of this study are applicable in developing a spectrum of fibrocartilaginous tissues for meniscal repair via oxygen tension mediated modulation of gene expression and tissue production.

## Conclusion

For the first time, we document the response of human MFCs isolated from inner and outer regions to 3D scaffold culture under normal and low oxygen tension. Human MFCs from either region expanded in monolayer and subsequently cultured on collagenous 3D scaffolds for 21 days demonstrate equivalent potential to redifferentiate toward a chondrogenic pattern of gene expression and tissue production in the presence of defined chondrogenic medium, following phenotypic loss associated with monolayer expansion. MFCs seeded in collagen scaffolds cultured under 21% O_2_ maintained greater potential to differentiate toward a fibrochondrocyte-like genotype, expressing significantly higher levels of aggrecan and COMP (p <0.05) compared to those scaffolds incubated in 3% O_2_ culture conditions. Additionally, normoxic cultured scaffolds expressed elevated collagen type II at levels approaching statistical significance compared to MFCs in hypoxic cultured scaffolds while depositing ECM richer in collagen type II and sulfated proteoglycan (p = 0.05). To put this in context for prospective meniscal tissue engineers, MFC from both inner and outer meniscus are equally suitable for fibrocartilaginous tissue production following serial monolayer expansion. Normoxic (21% O_2_) conditions drive increased production of collagen type II, aggrecan and COMP in 3D scaffold culture of MFCs. Investigators seeking to develop cell-based, functional meniscal implants should be mindful of the role of differential oxygen tension in modulating MFC mRNA transcript levels and ECM synthesis.

## Competing interests

The authors declare that they have no competing interests.

## Authors’ contributions

RC conducted all experiments and was responsible for data collection, analysis and drafting the manuscript. NJ was involved in study design, data analysis, supervision and edited the manuscript. HU was involved in study design, data analysis and edited the manuscript. AA was responsible for conception of the study, data analysis, supervision of the project and helped draft the manuscript. All authors read and approved the final manuscript.

## Pre-publication history

The pre-publication history for this paper can be accessed here:

http://www.biomedcentral.com/1471-2474/14/353/prepub

## References

[B1] Aagaard H Verdonk R Function of the normal meniscus and consequences of meniscal resection Scand J Med Sci Sports 1999 14 3 134 140 10380269 10.1111/j.1600-0838.1999.tb00443.x

[B2] McDermott ID Amis AA The consequences of meniscectomy J Bone Joint Surg Br 2006 14 12 1549 1556 17159163 10.1302/0301-620X.88B12.18140

[B3] Sanchez-Adams J The knee meniscus: a complex tissue of diverse cells Cell Mol Bioeng 2009 14 332 340 10.1007/s12195-009-0066-6

[B4] Chevrier A Nelea M Hurtig MB Hoemann CD Buschmann MD Meniscus structure in human, sheep, and rabbit for animal models of meniscus repair J Orthop Res 2009 14 9 1197 1203 10.1002/jor.20869 19242978

[B5] Nakata K Shino K Hamada M Mae T Miyama T Shinjo H Horibe S Tada K Ochi T Yoshikawa H Human meniscus cell: characterization of the primary culture and use for tissue engineering Clin Orthop Relat Res 2001 14 S208 218 11603705

[B6] Fukazawa I Hatta T Uchio Y Otani H Development of the meniscus of the knee joint in human fetuses Congenit Anom (Kyoto) 2009 14 1 27 32 10.1111/j.1741-4520.2008.00216.x 19243414

[B7] Arnoczky SP Warren RF Microvasculature of the human meniscus Am J Sports Med 1982 14 2 90 95 10.1177/036354658201000205 7081532

[B8] Arnoczky SP Warren RF The microvasculature of the meniscus and its response to injury. An experimental study in the dog Am J Sports Med 1983 14 3 131 141 10.1177/036354658301100305 6688156

[B9] King D The healing of semilunar cartilage J Bone Joint Surg 1936 14 333 342

[B10] Gu YL Wang YB Treatment of meniscal injury: a current concept review Chin J Traumatol 2010 14 6 370 376 21126396

[B11] Clayton RA Court-Brown CM The epidemiology of musculoskeletal tendinous and ligamentous injuries Injury 2008 14 12 1338 1344 10.1016/j.injury.2008.06.021 19036362

[B12] Berthiaume MJ Raynauld JP Martel-Pelletier J Labonte F Beaudoin G Bloch DA Choquette D Haraoui B Altman RD Hochberg M Meniscal tear and extrusion are strongly associated with progression of symptomatic knee osteoarthritis as assessed by quantitative magnetic resonance imaging Ann Rheum Dis 2005 14 4 556 563 10.1136/ard.2004.023796 15374855 PMC1755443

[B13] Roos H Lauren M Adalberth T Roos EM Jonsson K Lohmander LS Knee osteoarthritis after meniscectomy: prevalence of radiographic changes after twenty-one years, compared with matched controls Arthritis Rheum 1998 14 4 687 693 10.1002/1529-0131(199804)41:4<687::AID-ART16>3.0.CO;2-2 9550478

[B14] McNicholas MJ Pengas IP Assiotis A Nash W Hatcher J Banks J Total meniscectomy in adolescents: a 40-year follow-up J Bone Joint Surg Br 2012 14 12 1649 1654 23188906 10.1302/0301-620X.94B12.30562

[B15] Fairbank TJ Knee joint changes after meniscectomy J Bone Joint Surg Br 1948 14 4 664 670 18894618

[B16] Lee AS Kang RW Kroin E Verma NN Cole BJ Allograft meniscus transplantation Sports Med Arthrosc 2012 14 2 106 114 10.1097/JSA.0b013e318246f005 22555208

[B17] Zhang Z Arnold JA Williams T McCann B Repairs by trephination and suturing of longitudinal injuries in the avascular area of the meniscus in goats Am J Sports Med 1995 14 1 35 41 10.1177/036354659502300106 7726348

[B18] Longo UG Campi S Romeo G Spiezia F Maffulli N Denaro V Biological strategies to enhance healing of the avascular area of the meniscus Stem Cells Int 2012 14 528359 22220179 10.1155/2012/528359 PMC3246301

[B19] Arnoczky SP Warren RF Spivak JM Meniscal repair using an exogenous fibrin clot. An experimental study in dogs J Bone Joint Surg Am 1988 14 8 1209 1217 3417706

[B20] Kon E Filardo G Tschon M Fini M Giavaresi G Marchesini Reggiani L Chiari C Nehrer S Martin I Salter DM Tissue engineering for total meniscal substitution: animal study in sheep model–results at 12 months Tissue Eng Part A 2012 14 15–16 1573 1582 22500654 10.1089/ten.TEA.2011.0572

[B21] Moriguchi Y Tateishi K Ando W Shimomura K Yonetani Y Tanaka Y Kita K Hart DA Gobbi A Shino K Repair of meniscal lesions using a scaffold-free tissue-engineered construct derived from allogenic synovial MSCs in a miniature swine model Biomaterials 2013 14 9 2185 2193 10.1016/j.biomaterials.2012.11.039 23261221

[B22] Khoury MA Goldberg VM Stevenson S Demonstration of HLA and ABH antigens in fresh and frozen human menisci by immunohistochemistry J Orthop Res 1994 14 6 751 757 10.1002/jor.1100120602 7983550

[B23] Verdonk P Beaufils P Bellemans J Djian P Heinrichs EL Huysse W Laprell H Siebold R Verdonk R the Actifit Study G Successful treatment of painful irreparable partial meniscal defects with a polyurethane scaffold: two-year safety and clinical outcomes Am J Sports Med 2012 14 4 844 853 10.1177/0363546511433032 22328711

[B24] Gunja NJ Athanasiou KA Passage and reversal effects on gene expression of bovine meniscal fibrochondrocytes Arthritis Res Ther 2007 14 5 R93 10.1186/ar2293 17854486 PMC2212577

[B25] Adesida AB Grady LM Khan WS Hardingham TE The matrix-forming phenotype of cultured human meniscus cells is enhanced after culture with fibroblast growth factor 2 and is further stimulated by hypoxia Arthritis Res Ther 2006 14 3 R61 10.1186/ar1929 16563175 PMC1526627

[B26] Pereira H Frias AM Oliveira JM Espregueira-Mendes J Reis RL Tissue engineering and regenerative medicine strategies in meniscus lesions Arthroscopy 2011 14 12 1706 1719 10.1016/j.arthro.2011.08.283 22019234

[B27] Mueller SM Shortkroff S Schneider TO Breinan HA Yannas IV Spector M Meniscus cells seeded in type I and type II collagen-GAG matrices in vitro Biomaterials 1999 14 8 701 709 10.1016/S0142-9612(98)00189-6 10353653

[B28] Gruber HE Mauerhan D Chow Y Ingram JA Norton HJ Hanley EN Jr Sun Y Three-dimensional culture of human meniscal cells: extracellular matrix and proteoglycan production BMC Biotechnol 2008 14 54 10.1186/1472-6750-8-54 18582376 PMC2443126

[B29] Adesida AB Mulet-Sierra A Laouar L Jomha NM Oxygen tension is a determinant of the matrix-forming phenotype of cultured human meniscal fibrochondrocytes PLoS One 2012 14 6 e39339 10.1371/journal.pone.0039339 22720095 PMC3376130

[B30] Malda J Martens DE Tramper J van Blitterswijk CA Riesle J Cartilage tissue engineering: controversy in the effect of oxygen Crit Rev Biotechnol 2003 14 3 175 194 14743989

[B31] Farndale RW Buttle DJ Barrett AJ Improved quantitation and discrimination of sulphated glycosaminoglycans by use of dimethylmethylene blue Biochim Biophys Acta 1986 14 2 173 177 10.1016/0304-4165(86)90306-5 3091074

[B32] Adesida AB Grady LM Khan WS Millward-Sadler SJ Salter DM Hardingham TE Human meniscus cells express hypoxia inducible factor-1alpha and increased SOX9 in response to low oxygen tension in cell aggregate culture Arthritis Res Ther 2007 14 4 R69 10.1186/ar2267 17640365 PMC2206369

[B33] Khan WS Adesida AB Hardingham TE Hypoxic conditions increase hypoxia-inducible transcription factor 2alpha and enhance chondrogenesis in stem cells from the infrapatellar fat pad of osteoarthritis patients Arthritis Res Ther 2007 14 3 R55 10.1186/ar2211 17537234 PMC2206341

[B34] Murdoch AD Grady LM Ablett MP Katopodi T Meadows RS Hardingham TE Chondrogenic differentiation of human bone marrow stem cells in transwell cultures: generation of scaffold-free cartilage Stem Cells 2007 14 11 2786 2796 10.1634/stemcells.2007-0374 17656642

[B35] Livak KJ Schmittgen TD Analysis of relative gene expression data using real-time quantitative PCR and the 2(-Delta Delta C(T)) Method Methods 2001 14 4 402 408 10.1006/meth.2001.1262 11846609

[B36] Mauck RL Martinez-Diaz GJ Yuan X Tuan RS Regional multilineage differentiation potential of meniscal fibrochondrocytes: implications for meniscus repair Anat Rec (Hoboken) 2007 14 1 48 58 10.1002/ar.20419 17441197

[B37] Martin I Vunjak-Novakovic G Yang J Langer R Freed LE Mammalian chondrocytes expanded in the presence of fibroblast growth factor 2 maintain the ability to differentiate and regenerate three-dimensional cartilaginous tissue Exp Cell Res 1999 14 2 681 688 10.1006/excr.1999.4708 10585291

[B38] Barbero A Ploegert S Heberer M Martin I Plasticity of clonal populations of dedifferentiated adult human articular chondrocytes Arthritis Rheum 2003 14 5 1315 1325 10.1002/art.10950 12746904

[B39] Sekiya I Koopman P Tsuji K Mertin S Harley V Yamada Y Shinomiya K Nifuji A Noda M Dexamethasone enhances SOX9 expression in chondrocytes J Endocrinol 2001 14 3 573 579 10.1677/joe.0.1690573 11375127

[B40] de Crombrugghe B Lefebvre V Behringer RR Bi W Murakami S Huang W Transcriptional mechanisms of chondrocyte differentiation Matrix Biol 2000 14 5 389 394 10.1016/S0945-053X(00)00094-9 10980415

[B41] Sekiya I Tsuji K Koopman P Watanabe H Yamada Y Shinomiya K Nifuji A Noda M SOX9 enhances aggrecan gene promoter/enhancer activity and is up-regulated by retinoic acid in a cartilage-derived cell line, TC6 J Biol Chem 2000 14 15 10738 10744 10.1074/jbc.275.15.10738 10753864

[B42] Aigner T Gebhard PM Schmid E Bau B Harley V Poschl E SOX9 expression does not correlate with type II collagen expression in adult articular chondrocytes Matrix Biol 2003 14 4 363 372 10.1016/S0945-053X(03)00049-0 12935820

[B43] Duval E Leclercq S Elissalde JM Demoor M Galera P Boumediene K Hypoxia-inducible factor 1alpha inhibits the fibroblast-like markers type I and type III collagen during hypoxia-induced chondrocyte redifferentiation: hypoxia not only induces type II collagen and aggrecan, but it also inhibits type I and type III collagen in the hypoxia-inducible factor 1alpha-dependent redifferentiation of chondrocytes Arthritis Rheum 2009 14 10 3038 3048 10.1002/art.24851 19790048

[B44] Lafont JE Talma S Murphy CL Hypoxia-inducible factor 2alpha is essential for hypoxic induction of the human articular chondrocyte phenotype Arthritis Rheum 2007 14 10 3297 3306 10.1002/art.22878 17907154

